# Understanding period product use among young women in rural and urban India from a geospatial perspective

**DOI:** 10.1038/s41598-024-70383-w

**Published:** 2024-08-29

**Authors:** Sourav Biswas, Asraful Alam, Nazrul Islam, Ranjan Roy, Lakshminarayan Satpati

**Affiliations:** 1 Department of Population & Development, International Institute for Population Sciences, Mumbai, Maharashtra, 400088 India; 2 Department of Geography, Serampore Girls’ College, 13, T.C. Goswami Street, Serampore, Hooghly, West Bengal 712201 India; 3 Department of Geography, Cooch Behar Panchanan Barma University, Cooch Behar, West Bengal 736101 India; 4 Department of Geography and Applied Geography, University of North Bengal, P.O.-NBU, Darjeeling, West Bengal 734013 India; 5 Director, UGC-HRDC, University of Calcutta, Kolkata, West Bengal, 700019 India

**Keywords:** Menstrual hygiene, Young women, Reproductive health, NFHS-5, Regional disparities, Health care, Risk factors, Signs and symptoms

## Abstract

Ensuring proper menstrual hygiene management remains a significant challenge for young women in India. The term "exclusive use of hygienic period products during menstruation" refers to relying solely on period products like sanitary pads, tampons, or menstrual cups. Poor menstrual hygiene practices not only increase the risk of reproductive tract infections but also lead to various negative health outcomes, including discomfort and potential complications. This study explores factors associated with the exclusive use of period products during menstruation aged 15–24, investigates geographic disparities, examines rural–urban gaps, and assesses inequality in India. Utilizing data from the fifth National Family Health Survey (NFHS-5), responses from 2,41,180 women aged 15 to 24 were analysed using logistic regression and multivariate decomposition analyses to explore socioeconomic predictors. Moran's I statistics also assessed spatial dependency, while Lorenz curves and Gini coefficients measured inequality. Quintile and LISA maps visualized regional disparities. The study found that 76.15% of women in India reported exclusive use of hygienic period products during menstruation. Rural areas reported a lower percentage of exclusive use of hygienic period products (72.32%) during menstruation compared to urban areas (89.37%). Key factors associated with the exclusive use of hygienic period products among 15–24-year-old women in India include age, education, place of residence, wealth, access to media, and healthcare discussions. Geographically, central districts exhibited the lowest coverage (< 65%), while the Southern region reported the highest (> 85). The GINI coefficient of 0.39 highlighted moderate inequality in distribution. Decomposition analysis revealed that household wealth contributed 49.25% to rural–urban differences, followed by education (13.41%), media access (7.97%), and region (4.97%). This study highlights significant regional disparities and low utilization of hygienic period products among young women in India, particularly in central districts. Policymakers should prioritize interventions targeting these regions, addressing socio-economic disparities. Strategies to promote education, improve media access, and enhance household wealth can facilitate menstrual hygiene adoption. Initiatives to reduce sanitary napkin costs and increase accessibility, particularly in rural areas, are crucial to mitigating geographical disparities nationwide.

## Introduction

Menstruation, or the period, is a natural occurrence in the female body. It involves the shedding of the uterine lining that happens each month in women of reproductive age, which is usually from the early teenage years until menopause. Hormones play a crucial role in controlling this process, as they trigger the uterus to build up a lining of blood and tissue in anticipation of a potential pregnancy. In the absence of conception, the lining is released from the body through the vagina in the form of menstrual blood. While the typical menstrual cycle lasts around 28 days, it can vary among individuals^1–4^. Despite its natural occurrence, menstruation can be accompanied by discomfort or pain for some women. However, ensuring comprehensive menstrual health and hygiene (MHH), which encompasses the consistent and appropriate use of materials and facilities to ensure cleanliness, comfort, and health during menstruation, poses significant challenges, particularly in low- and middle-income countries (LMICs). Research indicates that over 50% of girls in LMICs do not adhere to adequate MHH practices^5–8^, with rural areas disproportionately associated. While empirical evidence suggests lower adoption rates of good MHH practices in rural areas compared to urban areas, it is crucial to understand the underlying barriers^9,10^. Apart from geographical disparities, factors such as awareness, affordability, and education play significant roles in shaping MHH behaviors^11–14^. Access to clean and absorbent materials, facilities for timely changing, cleaning, or disposal, and soap and water for personal hygiene are essential components of effective MHH. However, understanding and addressing the broader socio-economic, cultural, and infrastructural factors influencing MHH practices are paramount for safeguarding the health and well-being of girls and women worldwide, particularly adolescents who often face unique challenges due to limited access to information, resources, and social support systems.

Moreover, inadequate MHH is directly or indirectly associated with Sustainable Development Goals (SDGs) 3 (Good Health and Well-being), 4 (Quality Education), 5 (Gender Equality), and 6 (Clean Water and Sanitation). Achieving these goals is crucial for the overall development of young adolescents and their respective countries^10^. In India, menstruation is still stigmatized, and many myths and misunderstandings surround it, leading to untreated menstrual disorders, increased risk of reproductive tract infections due to poor menstrual hygiene practices, delayed diagnosis and management of menstrual-related health issues, and psychological distress stemming from societal taboos and discrimination associated with menstruation^15^. MHH remains a significant challenge for school-aged teenagers in India due to the lack of safe and sanitary facilities, as well as limited or no access to menstrual hygiene products and services. Consequently, many girls drop out of school, further exacerbating the issue. Adolescent girls in India face a widespread prevalence of menstrual-related issues^10^. These issues vary depending on the socio-cultural and regional factors, with 64% of girls experiencing at least one menstrual abnormality, such as irregular periods, heavy bleeding, or painful menstruation^16^. Poor menstrual hygiene and a lack of self-care are significant contributing factors to morbidity and related problems among girls aged 10–19. These problems include urinary tract infections, scabies in the vaginal area, atypical abdominal pain, absenteeism from school, and pregnancy complications^17^. Despite an estimated 113 million adolescent girls in India, roughly 68 million attend 1.4 million schools, with poor MHH practices and cultural taboos hindering their attendance^18,19^. Highlighting the intersectionality of MHH with human rights and sustainable development goals, Hennegan's systematic review underscores the importance of comprehensive interventions in reducing school absenteeism among adolescent girls^20^. Babbar et al. emphasize the multidimensional nature of achieving good menstrual health and propose action points, including creating an enabling sociocultural environment and providing adequate sanitation facilities^21^. Additionally, Sommer et al. stress the necessity of integrating MHH into emergency response efforts, particularly in addressing menstruation-related needs during crises, underscoring the urgency of addressing MHH comprehensively to ensure the well-being and rights of adolescent girls in India and globally^22^.

Menstrual abnormalities and disorders have physical, mental, social, psychological, and reproductive implications, affecting the daily lives of adolescents and their families. These problems lead to various psychosocial issues such as anxiety^23^. To address these challenges, it is essential to promote good MHH practices and awareness among adolescent girls in India. Additionally, access to affordable menstrual products, toilets, and handwashing facilities can improve the overall health and well-being of girls^24^. Schools can play a crucial role in providing such facilities and promoting MHH practices, creating a conducive environment for girls to learn and thrive^18,19^. Overall, addressing menstrual-related problems is crucial to achieving gender equality and ensuring that adolescent girls in India have the opportunity to reach their full potential^25,26^.

The Government of India recognizes the importance of promoting good menstrual hygiene practices among adolescent girls and women, as it is critical to their overall health and well-being. The government has undertaken several activities to raise awareness and provide access to affordable menstrual hygiene products^27–30^. In August 2011, a scheme was introduced to provide subsidized sanitary napkins to adolescent girls in rural areas, as their reproductive health decisions can impact future generations and their communities^24^. Menstrual Hygiene Day is also observed on May 28 each year to raise awareness of the challenges faced by women and girls during menstruation and promote solutions to address these problems. Despite concerted governmental efforts, there persists a substantial deficit in both the comprehension of the menstrual cycle and the adoption of appropriate hygiene protocols among adolescent girls, culminating in suboptimal menstrual hygiene practices^31^. Notably, various government schemes aim to promote menstrual hygiene. However, data from the most recent round of the National Family Health Survey (NFHS-5) indicate that the use of hygienic period products during menstruation is still lower in rural areas than in urban areas^28,30,32^. This highlights the need for increased efforts to promote awareness and access to affordable menstrual hygiene products in rural areas to improve the overall health and well-being of adolescent girls and women in India. Additionally, educational initiatives aimed at breaking the taboo and stigma around menstruation can help dispel myths and promote good menstrual hygiene practices^27,28,32,33^.

The existing literature, represented by studies from Babbar et al.^34^, Singh and Chakrabarty^35^, Singh et al.^32,33^, and Chakrabarty et al.^36^, has made significant strides in understanding various aspects of menstrual hygiene practices, including socio-demographic factors and geographic disparities. Singh et al.^32,33^ revealed factors associated with the exclusive use of hygienic period products during menstruation among rural adolescent women in India, offering insights into state- and district-level disparities to inform evidence-based policymaking and program design^32^. Singh and Chakrabarty^35^ pinpointed districts in India where urban women aged 15–24 face significant deficiencies in hygienic material use, addressing spatial disparities and providing policymakers with up-to-date evidence to combat regional inequalities^35^. Babbar et al. focused on socio-demographic factors influencing the choice of modern period products among girls and women, emphasizing the disparities between disposable and sustainable options and addressing period poverty and ecological concerns^34^. Chakrabarty et al. highlighted differences in focus and methodology, primarily investigating rural–urban gap variations across regions and socioeconomic groups^36^. However, a gap persists in the literature concerning detailed spatial variations and rural–urban disparities in hygienic period product use at both state and district levels. Recognizing this gap, our study aims to build upon existing research by exploring factors associated with the exclusive use of hygienic period products among women aged 15–24 in India. This demographic is pivotal as it spans adolescence to early adulthood, a critical period marked by significant physical, emotional, and social changes, including the onset of menstruation and the establishment of menstrual hygiene practices. Interventions during this age can profoundly shape lifelong menstrual health behaviors. Moreover, this demographic often faces barriers such as limited access to resources and educational opportunities, making them particularly susceptible to inadequate menstrual hygiene management. Therefore, targeting this age group is strategic for promoting sustainable menstrual health practices and mitigating associated challenges effectively. Our research aims to examine spatial patterns and clustering of hygienic period product use, identifying the degree of inequality at the state level and determining the rural–urban gap's contribution to observed differences in hygienic period product use during menstruation. By addressing these objectives, our study aims to contribute to a more comprehensive understanding of menstrual hygiene practices, facilitating the development of targeted interventions to improve women's health outcomes in India.

## Data and methods

### Data

**Fig. 1:**
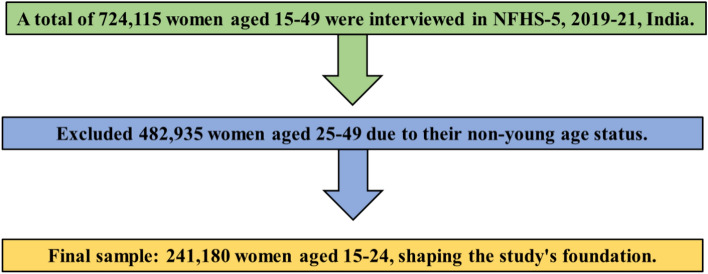
Flow chart showing the steps to select a representative sample of Indian young women aged 15–24 for the current study.

This study utilized data from the fifth National Family Health Survey (NFHS-5) conducted between 2019 to 2021, which is a nationally representative cross-sectional survey designed to collect data on various aspects of health in India. The survey collected demographic, socioeconomic, maternal and child welfare, reproductive health, and family planning data from 724,115 women and 101,839 men from 636,669 households in 28 states and 8 Union territories (UTs) across 707 districts. For this study, the data from 2,41,180 women aged 15 to 24 from 28 states and 8 UTs were analysed (see Fig. 1).

### Conceptual framework

**Fig. 2:**
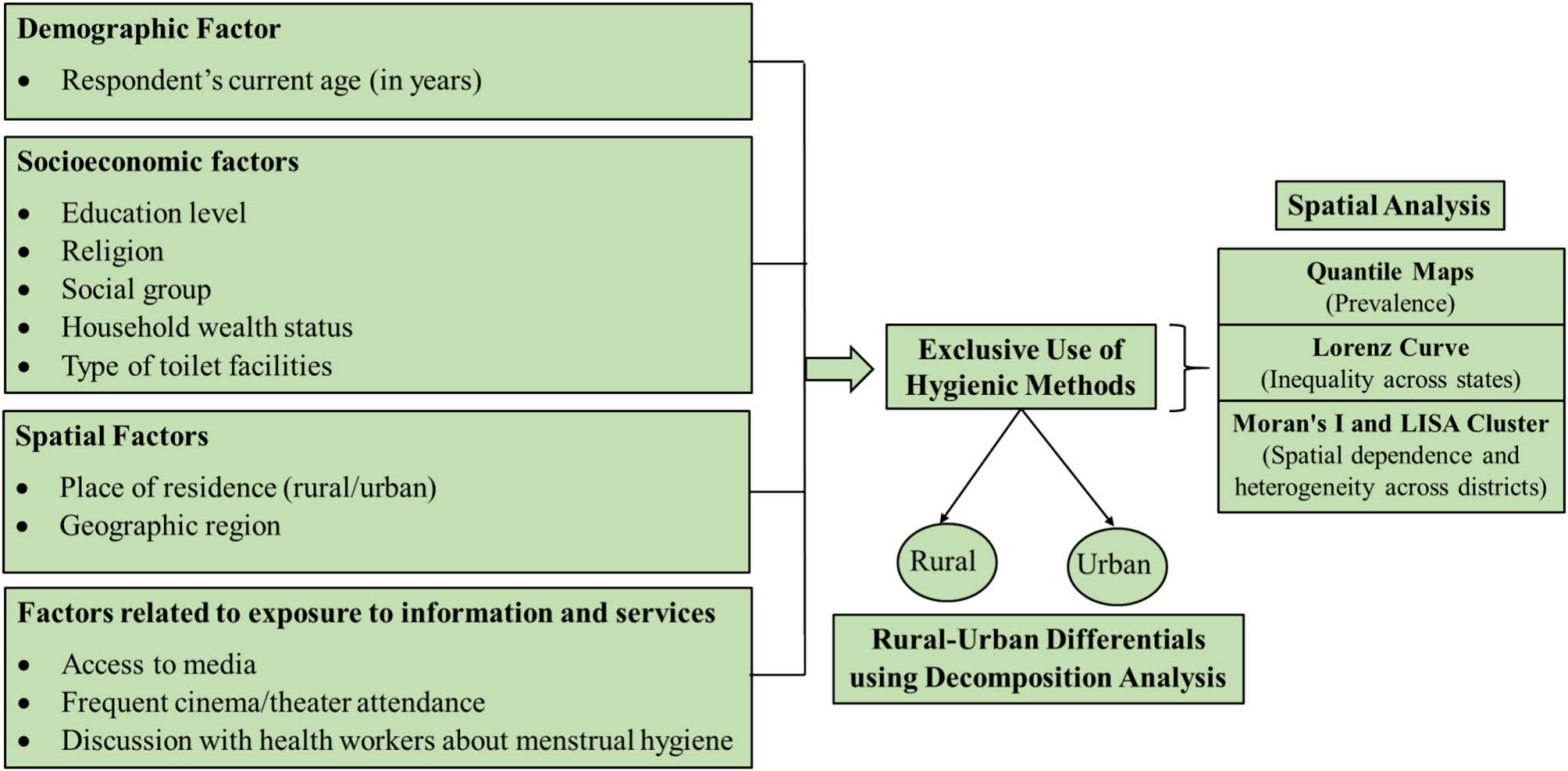
Conceptual framework of the study.

The analysis for this study is informed by a framework adapted from existing literature on period products during menstruation^32,34–40^. This framework delineates the paths by which different factors are associated with young Indian women's exclusive use of hygienic period products. It was proposed that characteristics linked to demographics, socioeconomics, geography, and exposure to information and services would correlate with the exclusive use of hygienic period products. Figure 2 shows the conceptual framework that directed the analysis for this investigation.

### Variables description

#### Outcome variable

This study focuses on the outcome variable of using period products during menstruation. The NFHS-5 survey posed a question to young women aged 15 to 24 regarding the methods they utilize for menstrual protection. Collecting data on menstrual hygiene can present challenges such as potential underreporting or overreporting of certain period products due to social desirability bias or recall bias. For instance, some women might underreport the use of less accepted or traditional methods like cloth due to stigma, while others might overreport the use of commercially available products like sanitary napkins to align with perceived societal expectations^41^. To ensure a reliable data collection process, the NFHS-5 survey employs female interviewers who administer questions on menstrual hygiene and private matters, aiming to create a safe and comfortable environment for respondents. Interviews were conducted in a private space, with no one allowed to be present during the survey, further enhancing the confidentiality and integrity of the data collected. The question asked is, "Women use different methods of protection during their menstrual period to prevent bloodstains from becoming evident. What do you use for protection?" The provided options include (i) cloth, (ii) locally-made napkins, (iii) sanitary napkins, (iv) tampons, (v) menstrual cups, (vi) nothing, and (vii) others. Based on the answers provided by women, we categorized the methods into hygienic and unhygienic categories. Hygienic period products encompassed women who reported using locally prepared napkins, sanitary napkins, tampons, and menstrual cups during their menstrual period (coded as '1'). Conversely, unhygienic period products included all other reported methods such as cloth, any other methods, and women who reported using nothing during their menstrual period (coded as '0'). The framework used to categorize these methods is based on previous literatures^32,42,43^.

#### Explanatory variables

The analysis encompassed various relevant socioeconomic and demographic factors, including the respondent's age group, place of residence, education, religion, caste, household wealth status, type of toilet, access to media, frequency of cinema/theatre visits, and discussing menstrual hygiene with healthcare workers. These independent variables were selected based on prior research on MHH and their availability in the NFHS-5 dataset^32,36,38,42^. The household wealth index served as a proxy indicator to assess the standard of living, determined from household asset ownership, housing characteristics, and access to necessities. The wealth index calculation method, employing principal component analysis (PCA) on household asset data, was provided in the NFHS-5 dataset. Exposure to mass media was gauged by the frequency of reading newspapers, watching television, and listening to the radio.

### Statistical analysis

The study initially employed bivariate analysis techniques, including the Chi-square test, to elucidate disparities in the exclusive use of hygienic period products during menstruation aged 15 to 24 in India, based on their socioeconomic predictors. To assess the relationship between the outcome and each predictor variable, the study utilized unadjusted and adjusted logistic regressions, with significance set at < 0.05. Unadjusted odds ratios (UORs) and adjusted odds ratios (AORs) with 95% confidence intervals (CIs) were reported.

Multivariate decomposition analysis was conducted to discern the contributions of covariates explaining group differences to average predictions, aiming to identify characteristics contributing to differences in hygienic period products use between urban and rural residents. This analysis decomposed observed differences into compositional differences (endowments) 'E' and effects of characteristics differing in coefficients or behavioural change 'C' responses for selected predictor variables. For instance, a characteristic could pertain to a woman's level of education, with its effect on how education influences her access to hygienic menstrual products. Inclusion of such examples would aid in elucidating the analysis within the context of MHH practices and the models used in the paper. In the nonlinear model, the dependent variable is a function of a linear combination of predictors and regression coefficients:

$${\text{Y }} = {\text{ F}}\left( {{\text{X}}\upbeta } \right) \, = {\text{ e}}\left( {{\text{X}}\upbeta } \right)/\left( {{1} + {\text{ e}}\left( {{\text{X}}\upbeta } \right)} \right)$$

where Y denotes the n*1 dependent variable vector, X an n*K matrix of independent variables and β a K*1 vector of coefficients. The proportion difference in Y between urban A and rural B of the exclusive use of hygienic period products can be decomposed as:

$${\text{Y}}_{{\text{A}}} - {\text{ Y}}_{{\text{B}}} = {\text{ F}}\left( {{\text{X}}_{{\text{A}}} \upbeta_{{\text{A}}} } \right) \, - {\text{ F}}\left( {{\text{X}}_{{\text{B}}} \upbeta_{{\text{B}}} } \right)$$

For the log odds of the exclusive use of hygienic period products, the proportion of the model is written as.

The component ‘E’ is the difference attributable to endowment change, usually called the explained component. The ‘C’ component is the difference attributable to coefficient (behavioural) change, usually called the unexplained component. The model structure for the decomposition analysis was:

$${\text{Logit}}\left( {\text{A}} \right) \, - {\text{ Logit}}\left( {\text{B}} \right) \, = \, \left[ {\upbeta_{{0{\text{A}}}} - \, \upbeta_{{0{\text{B}}}} } \right] \, + \, \sum \upbeta_{{{\text{ijA}}}} \left[ {{\text{X}}_{{{\text{ijA}}}} - {\text{ X}}_{{{\text{ijB}}}} } \right] \, + \, \sum {\text{X}}_{{{\text{ijB}}}} \left[ {\upbeta_{{{\text{ijA}}}} {-} \, \upbeta_{{{\text{ijB}}}} } \right]$$

where β_0A_ is the intercept in the regression equation for urban, β_0B_ is the intercept in the regression equation for rural, β_ijA_ is the coefficient of the jth category of the ith determinant for urban, β_ijB_ is the coefficient of jth category of the ith determinant for rural, X_ijA_ is the proportion of the jth category of the ith determinant for urban, and X_ijB_ is the proportion of the jth category of the ith determinant for rural. The command mvdcmp was used to perform multivariate decomposition analysis in STATA 16.1.

The Lorenz curve visually represented the inequality of hygienic period products use during menstruation between Indian States and Union Territories, with the Gini coefficient quantifying this inequality. Max O. Lorenz developed it in 1905 to show wealth inequality. The X-axis represents the cumulative percentage of young women aged 15–24, while the Y-axis represents the cumulative percentage of young women aged 15–24 using hygienic period products during menstruation.

The formula for the Lorenz Curve is:

$$L\left(x\right)=\frac{{\sum }_{i=1}^{n}{\text{y}}_{i}}{nY}$$

where $L$($x$): is the Lorenz Curve value at a given point on the X-axis, $n$: is the total number of observations, ${\text{y}}_{i}$: is the cumulative percentage of young women aged 15–24 using hygienic period products during menstruation at the $ith$ observation, and $Y$: is the total percentage of young women aged 15–24.

The Gini coefficient is a numerical measure of inequality that complements the Lorenz Curve. It quantifies the degree of inequality in the distribution of hygienic period products usage during menstruation among young women aged 15–24. A flat curve would indicate more equitable distribution, while a bowed-out curve signifies greater inequality. Where the value ranges between 0 and 1. 1 means perfect inequality, and 0 means perfect equality.

The formula for the Gini coefficient is:

$$G=\frac{{\sum }_{i=1}^{n}{\sum }_{j=1}^{n}|{x}_{i}-{x}_{j}|}{2{n}^{2}\overline{x} }$$

where $G$: is the Gini coefficient, $n$: is the total number of observations, ${x}_{i}$ and ${x}_{j}:$ are the cumulative percentage of young women aged 15–24 using hygienic period products during menstruation at the $ith$ and $jth$ observations respectively, $\overline{x }$: is the mean of the cumulative percentages.

We employed spatial analysis techniques to investigate the spatial dependence of exclusive use of hygienic period products during menstruation across Indian districts. One of the primary tools utilized was the Local Indicators of Spatial Association (LISA) analysis, which allows for identifying spatial clusters and outliers in the data. This analysis is particularly useful for understanding how neighbouring districts influence each other's hygienic period product usage patterns. The LISA cluster map visually represents these spatial patterns, categorizing districts into four groups: "high-high," "low-low," "low–high," and "high-low" based on their hygienic period product usage rates relative to neighbouring districts. The "high-high" clusters represent districts with consistently high rates of hygienic period product usage, while "low-low" clusters denote districts with consistently low rates. Conversely, "low–high" clusters indicate districts with low usage rates surrounded by neighbours with high rates, and "high-low" clusters represent districts with high usage rates surrounded by neighbours with low rates. We calculated the Univariate Local Moran's I statistic for each district to quantify the degree of spatial autocorrelation. This statistic measures the extent to which the usage of the hygienic period product in a given district is correlated with the usage in neighbouring districts. To establish the spatial connections among districts, we created a spatial weight matrix using the queen contiguity method^44^. This method defines spatial relationships based on shared borders, ensuring a comprehensive representation of district-to-district connections. The spatial weight matrix (${w}_{ij}$) for Univariate Local Moran's I is given by:

$${w_{ij}} = \left\{ {\begin{array} {ll} 1 & \quad {{\text{if}}\;{\text{districts}}\;{\text{i}}\;{\text{and}}\;{\text{j}}\;{\text{share}}\;{\text{a}}\;{\text{border}}} \\ 0 & \quad {{\text{otherwise}}} \\ \end{array} } \right.$$

Each of the 707 Indian districts was uniquely identified by a code, and the spatial weight matrix captured the geographical interdependencies among them. The choice of the queen contiguity method was deliberate, as it accounts for shared borders and provides a well-rounded spatial weight matrix for subsequent analyses. Significance in our spatial analysis was determined at the 0.05 level for Local Moran's I statistics and for LISA significance maps. This significance level was chosen to ensure robustness in identifying spatial patterns and relationships, allowing us to draw meaningful conclusions about the spatial dynamics of exclusive use of hygienic period product during menstruation across the diverse landscape of India. Moran's I calculations were performed utilizing the following formulas:

$$\text{Univariate Local Moran{{'}}s I }\left({I}_{i}\right)=\frac{({x}_{i}- \overline{x }) ({\sum }_{j=1}^{n}{w}_{ij }({x}_{i}- \overline{x }))}{\sqrt{{\sum }_{j=1}^{n}{{(x}_{j}- \overline{x })}^{2}{\sum }_{j=1}^{n}{{(x}_{j}- \overline{x } )}^{2}}}$$

where ${x}_{i}:$ is the value of hygienic period products during menstruation in district $i$, $\overline{x }$: is the mean of hygienic period products during menstruation across all districts, ${w}_{ij}:$ is the spatial weight between districts $i$ and $j$, and the sums are over all neighbouring features $j$ of feature $i$.

### Mapping

Quintile maps are utilized as a means of visually presenting the distribution of exclusive use of period products during menstruation among various districts and states in India. These maps categorize regions into five groups or quintiles based on the prevalence of exclusive use of period products, with each quintile represented by distinct shading. Quintile maps were generated using QGIS (Version 2.18.25) software (https://qgis.org/en/site/). Additionally, GeoDa software (https://geodacenter.github.io/download.html) was employed to produce LISA cluster map, significance map and Moran's Scatter Plot.

### Ethics approval and consent to participate

The study used data freely available at https://www.dhsprogram.com and does not contain any personally identifiable information.

## Result

### Exclusive use of hygienic period products by background characteristics

**Fig. 3:**
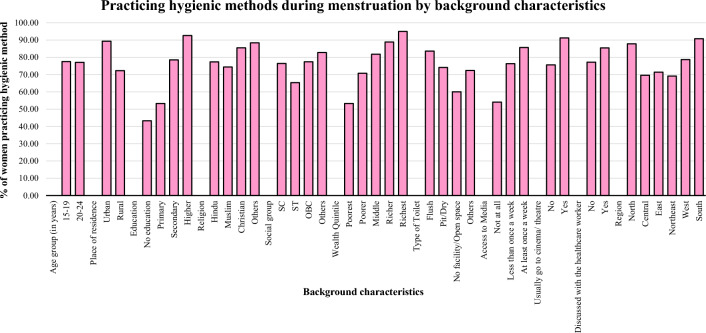
Exclusive use of hygienic period products by background characteristics among women aged 15–24 years in India, NFHS-5, 2019–2021.

Figure 3 illustrates that 76.15% of women aged 15 to 24 in India reported exclusive use of hygienic period products during menstruation. The percentage of women exclusively employing hygienic period products varied significantly across different background characteristics. Rural areas showed a lower percentage of exclusive hygienic period product usage during menstruation (72.32%) than urban areas (89.37%). Women's percentage of exclusive hygienic period product usage during menstruation having no education reported the lowest percentage (43.27%), while highly educated women reported the highest (92.63%) percentage. This discrepancy between the two groups amounted to nearly 49%. The lowest percentage of exclusive hygienic period product usage during menstruation was reported among Muslim women (74.4%), while the highest was among Christian women (85.52%). Marginalized social groups ST (65.36%), SC (76.43%) and OBC (77.41%) reported lower percentages of exclusive hygienic period product usage during menstruation, while other social groups (82.82%) exhibited higher percentages. Significant disparities were observed in exclusive hygienic period product usage during menstruation between the poorest and richest wealth categories, with only 53.29% of women in the poorest category utilizing exclusive methods compared to 94.98% of those in the richest category. Furthermore, women lacking toilet facilities or open spaces reported a lower percentage (60.05%) of exclusive hygienic period product usage during menstruation, while those with flush toilet facilities exhibited 83.62%, and those with pit/dry toilet facilities demonstrated 74.15%. Access to media at least once a week (85.69%) was associated with a higher percentage of exclusive use of hygienic period products during menstruation, while only 54.07% of women who had no access to media at all utilized hygienic period products during menstruation. Regular visits to cinema/theatre at least once a month (91.25%) were linked to higher hygienic period product usage, yet only 75.62% of women who usually did not go to such venues employed hygienic period products during menstruation. Furthermore, discussions on menstrual hygiene with healthcare workers (85.5%) were associated with increased exclusive use of hygienic period products, although only 77.19% of women who had not engaged in such discussions utilized hygienic period products during menstruation. Regional disparities were also observed, with the Northeast (69.13%), Central (69.63%), and Eastern (71.43%) regions reporting lower percentages of exclusive hygienic period products usage during menstruation, while the Southern (90.71%) region reported the highest percentage; in addition, the Northern region exhibited 87.83% and the Western region 78.67% utilization rates of hygienic period products during menstruation.

### Geographic distribution of exclusive use of hygienic period products during menstruation among 15–24 year-old women across 28 states, 8 UTs, and 707 districts in India

**Fig. 4:**
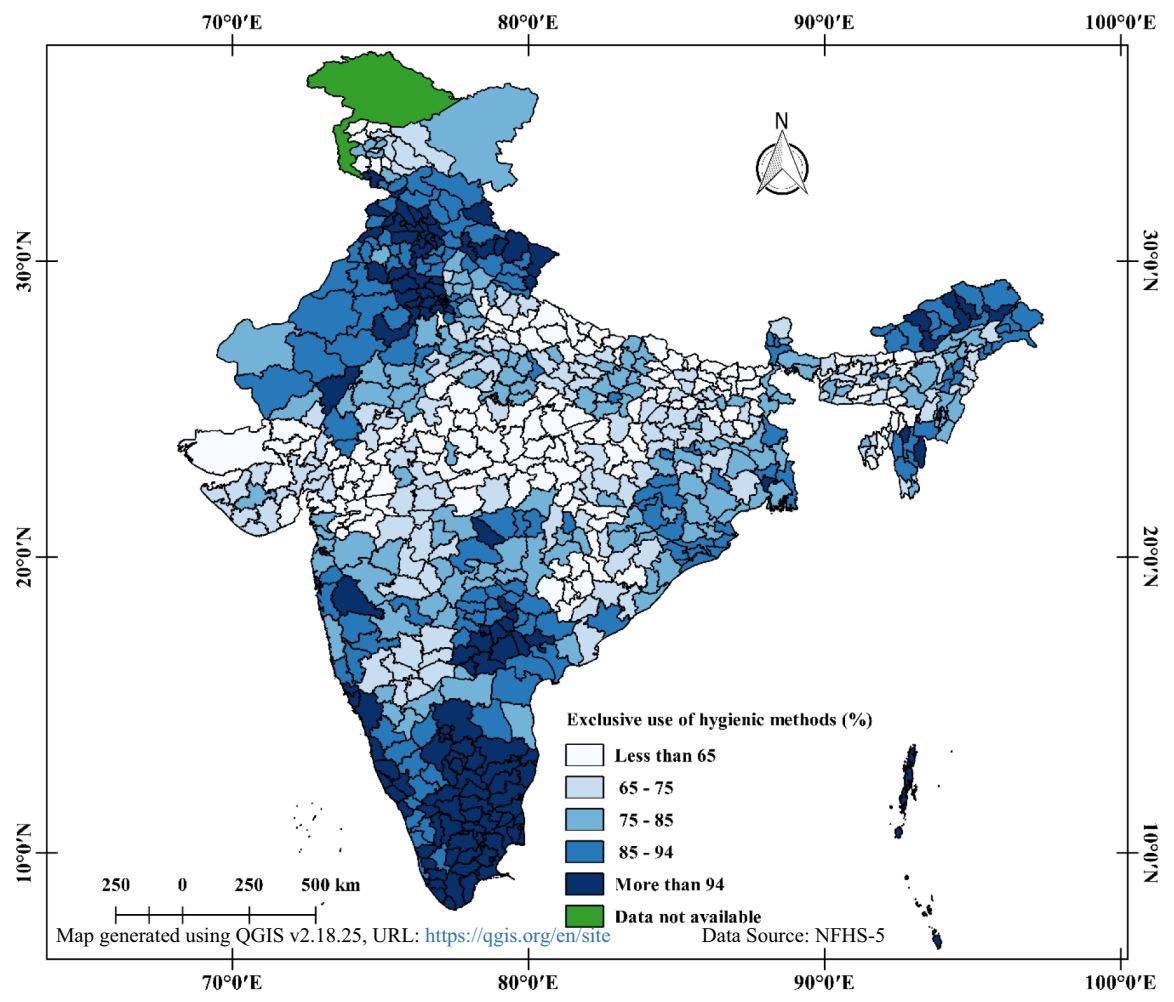
District-wise distribution of practicing hygienic period products during menstruation aged 15–24 years in India, NFHS-5, 2019–2021.

When analysing the exclusive use of hygienic period products among 15 to 24-year-old women in India, it's important to consider the regional differences. By examining state and district-level data, we can gain a better understanding of the spatial patterns and heterogeneity of this issue across the country. Figure 4 provides insight into the district-level spatial pattern of exclusive use of hygienic period products. The worst situation was found in the central districts of India, where the majority had less than 65% coverage of exclusive use of hygienic period products. Similarly, most districts in Uttar Pradesh and Bihar also had less than 65% coverage. The north-eastern districts varied from poor to good coverage, with Assam and Meghalaya having less than 65% coverage in one-third of their areas, while the rest states had high coverage (more than 75%). In contrast, most Northern districts had high coverage (more than 75%), with the exception of the western district of Jammu & Kashmir (less than 65%). Southern districts showed excellent coverage (more than 85%) of exclusive use of hygienic period products.

**Fig. 5:**
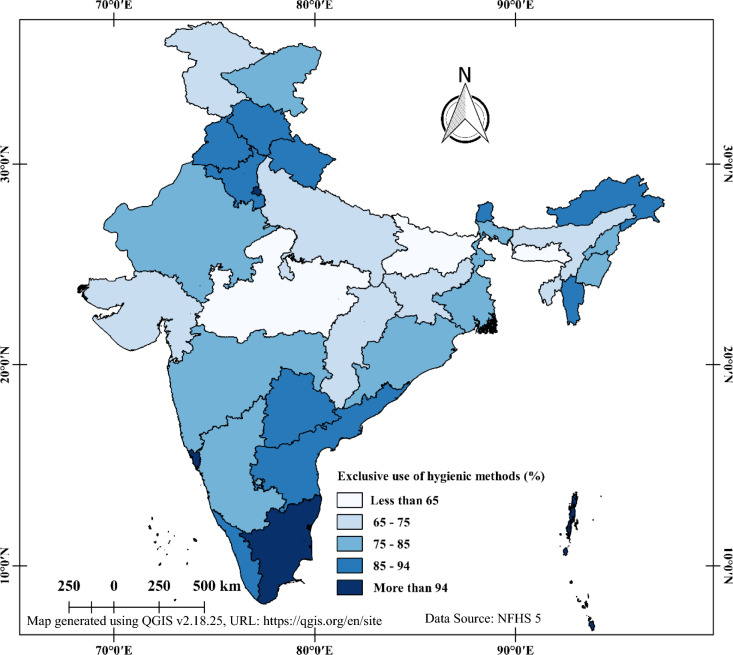
State-wise distribution of practicing hygienic period products during menstruation aged 15–24 years in India, NFHS-5, 2019–2021.

Figure 5 illustrates the state-wise variation in the exclusive use of hygienic period products among 15 to 24-year-old women in India. Bihar, Madhya Pradesh, and Meghalaya had the lowest prevalence of exclusive use of hygienic period products (less than 65%). Jammu & Kashmir, Uttar Pradesh, Jharkhand, Chhattisgarh, Gujarat, and Assam had 65 to 75% coverage, while Himachal Pradesh, Uttarakhand, Punjab, Haryana, Arunachal Pradesh, Mizoram, Sikkim, Telangana, Andhra Pradesh, and Kerala had 85 to 94% coverage. Tamil Nadu was the only state with more than 94% coverage of exclusive use of hygienic period products.

### Factors associated with exclusive use of hygienic period products

The logistic regression model was employed to investigate the factors of exclusive use of hygienic period products aged 15–24 in India. Table 1 displays the odds ratios derived from the model, shedding light on the factors associated with the exclusive use of hygienic period products. The logistic regression analysis results indicate that women aged 20–24 years are approximately 0.84 times less likely (AOR: 0.84, 95% CI 0.82–0.86) to use hygienic period products compared to their 15–19 years old counterparts. Additionally, women residing in rural areas are approximately 0.74 times less likely (AOR: 0.74, CI 0.72–0.77) to use hygienic period products than urban women. Conversely, women with higher education have approximately 5.38 times higher likelihood (AOR: 5.38, 95% CI 5.11–5.66) of using hygienic period products than those without education. Muslim women are approximately 0.72 times less likely (AOR: 0.72, CI 0.70–0.75) to use exclusive hygienic period products, while Christian women have approximately 1.49 times higher likelihood (AOR: 1.49, CI 1.36–1.63) compared to Hindu women. Similarly, ST women have approximately 0.74 times lower likelihood (AOR: 0.74, CI 0.71–0.76), OBC women have approximately 0.95 times lower likelihood (AOR: 0.95, CI 0.93–0.98), and women belonging to other social categories have approximately 1.20 times higher likelihood (AOR: 1.20, CI 1.16–1.24) of using hygienic period products compared to SC women. Women from the richest households are approximately 5.29 times more likely (AOR: 5.29, CI 4.99–5.61) to use hygienic period products compared to the poorest women. Moreover, women without toilet facilities or open spaces are approximately 0.82 times less likely (AOR: 0.82, CI 0.79–0.84) to use hygienic period products exclusively compared to women with flush toilet facilities. Furthermore, women who have access to media at least once a week have approximately 1.80 times higher likelihood (AOR: 1.80, CI 1.75–1.85) of using hygienic period products compared to those who do not have access to any media. Similarly, women who usually go to cinema/theatre at least once a month have approximately 1.45 times higher likelihood (AOR: 1.45, CI 1.39–1.52) of using hygienic period products than those who do not go to cinema/theatre at least once a month. Women who discussed menstrual hygiene with healthcare workers are approximately 1.47 times more likely (AOR: 1.47, CI 1.33–1.62) to use hygienic period products compared to those who did not have any discussions with healthcare workers. Western, Central, North-eastern, and Eastern regions of India have approximately 0.45 times, 0.47 times, 0.52 times, and 0.70 times lower likelihood, respectively, of using hygienic period products compared to the Northern region. Conversely, the Southern region has approximately 1.04 times higher likelihood of using hygienic period products compared to the Northern region.

**Table 1:** Odds ratio for practicing hygienic period products during menstruation by background characteristics of young women in India, NFHS-5 (2019–2021).

Background characteristics	Model 1 OR (CI)	Model 2 OR (CI)	Model 3 OR (CI)
Age group (in years)			
15–19^®^	1 [1.00, 1.00]	1 [1.00, 1.00]	1 [1.00, 1.00]
20–24	0.87*** [0.85, 0.89]	0.91*** [0.89, 0.93]	0.84*** [0.82, 0.86]
Place of residence			
Urban^®^		1 [1.00, 1.00]	1 [1.00, 1.00]
Rural		0.50*** [0.48, 0.51]	0.74*** [0.72, 0.77]
Education			
No education^®^	1 [1.00, 1.00]		1 [1.00, 1.00]
Primary	1.40*** [1.34, 1.47]		1.25*** [1.19, 1.31]
Secondary	3.64*** [3.52, 3.78]		2.58*** [2.48, 2.68]
Higher	10.04*** [9.55, 10.55]		5.38*** [5.11, 5.66]
Religion			
Hindu^®^		1 [1.00, 1.00]	1 [1.00, 1.00]
Muslim		0.72*** [0.70, 0.75]	0.87*** [0.84, 0.90]
Christian		1.49*** [1.36, 1.63]	1.37*** [1.25, 1.51]
Others		1.73*** [1.59, 1.89]	1.48*** [1.35, 1.62]
Social group			
SC^®^	1 [1.00, 1.00]	1 [1.00, 1.00]	1 [1.00, 1.00]
ST	0.74*** [0.71, 0.76]	0.74*** [0.71, 0.77]	0.85*** [0.82, 0.89]
OBC	0.95*** [0.93, 0.98]	1.07*** [1.04, 1.10]	0.91*** [0.88, 0.93]
Others	1.20*** [1.16, 1.24]	1.42*** [1.38, 1.47]	1.08*** [1.05, 1.12]
Wealth quintile			
Poorest^®^			1 [1.00, 1.00]
Poorer			1.52*** [1.47, 1.56]
Middle			2.09*** [2.02, 2.16]
Richer			2.95*** [2.82, 3.08]
Richest			5.29*** [4.99, 5.61]
Type of toilet			
Flush^®^	1 [1.00, 1.00]	1 [1.00, 1.00]	1 [1.00, 1.00]
Pit/dry	0.73*** [0.70, 0.76]	0.83*** [0.80, 0.87]	1.02 [0.97, 1.06]
No facility/open space	0.46*** [0.44, 0.47]	0.50*** [0.49, 0.51]	0.82*** [0.79, 0.84]
Others	0.73*** [0.71, 0.76]	0.84*** [0.81, 0.86]	1.10*** [1.06, 1.14]
Access to media			
Not at all^®^		1 [1.00, 1.00]	1 [1.00, 1.00]
Less than once a week		2.17*** [2.11, 2.23]	1.59*** [1.55, 1.64]
At least once a week		2.97*** [2.90, 3.05]	1.80*** [1.75, 1.85]
Usually go to cinema/theatre at least once in a month			
No^®^	1 [1.00, 1.00]		1 [1.00, 1.00]
Yes	1.96*** [1.87, 2.05]		1.45*** [1.39, 1.52]
Discussed menstrual hygiene with the healthcare worker			
No^®^		1 [1.00, 1.00]	1 [1.00, 1.00]
Yes		1.47*** [1.34, 1.62]	1.47*** [1.33, 1.62]
Region			
North^®^	1 [1.00, 1.00]	1 [1.00, 1.00]	1 [1.00, 1.00]
Central	0.35*** [0.34, 0.37]	0.42*** [0.41, 0.44]	0.47*** [0.45, 0.49]
East	0.43*** [0.41, 0.45]	0.52*** [0.50, 0.54]	0.70*** [0.67, 0.73]
Northeast	0.32*** [0.30, 0.34]	0.39*** [0.37, 0.42]	0.52*** [0.48, 0.55]
West	0.46*** [0.44, 0.48]	0.47*** [0.45, 0.50]	0.45*** [0.43, 0.47]
South	1.11*** [1.06, 1.17]	1.16*** [1.10, 1.21]	1.04** [0.99, 1.10]

*OR* odds ratio, *CI* confidence interval, *®* reference category.

***Significant at 1%, **Significant at 5%, *Significant at 10%.

### Inequality in exclusively using hygienic period products during menstruation across Indian States

**Fig. 6:**
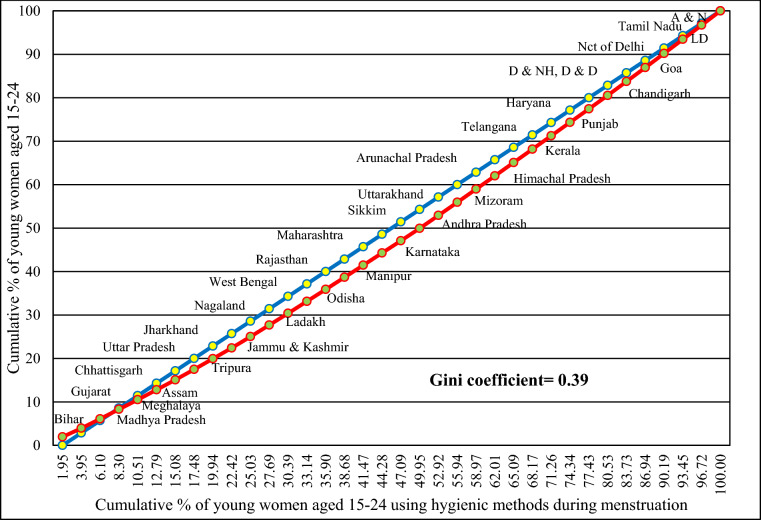
Inequality in practicing hygienic period products during menstruation across Indian States using the Lorenz curve, NFHS-5, 2019–2021.

Figure 6 provides a visual representation of the disparities in the adoption of menstrual hygiene practices among young women aged 15–24 across Indian States and Union Territories, as delineated by the Lorenz curve. The X-axis illustrates the cumulative percentage of young women aged 15–24, while the Y-axis represents the cumulative percentage of young women aged 15–24 using hygienic period product during menstruation. The Gini coefficient serves as a metric of inequality, discerning the extent of disparity in menstrual hygiene practices across these areas. A Gini coefficient of 0 represents perfect equality where everyone has the same level of hygienic period product usage, while a coefficient of 1 signifies complete inequality where one entity possesses all the hygienic period product usage. Our analysis yielded a coefficient of 0.39, indicating a moderate level of inequality in hygienic period product usage among different States and UTs.

### Spatial dependence and heterogeneity of exclusively using hygienic period products during menstruation across Indian districts

**Fig. 7:**
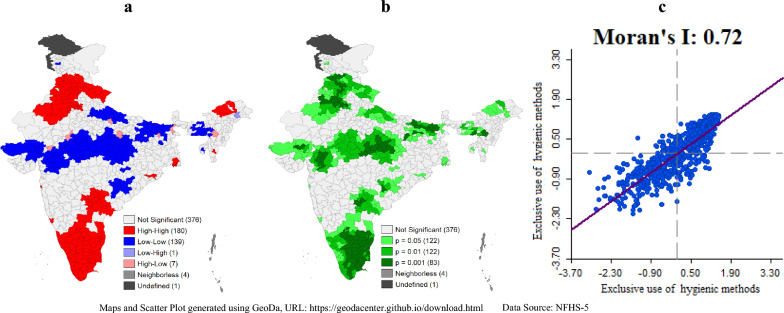
(**a**) Local spatial pattern (LISA map), (**b**) significance map, and (**c**) Moran's Scatter Plot showing spatial relationships and patterns of practicing hygienic period products during menstruation in Indian districts, NFHS-5, 2019–2021.

The LISA cluster map in Fig. 7a shows that there are 180 districts in the north (Rajasthan, Punjab, Haryana, Himachal Pradesh), northeast (Arunachal Pradesh), and southern (Andhra Pradesh, Telangana, Tamil Nadu, Karnataka, Kerala, and Goa) regions of India that have relatively higher levels of coverage of exclusive use of hygienic period products during menstruation compared to surrounding districts (high-high zone). In contrast, 139 districts in the north and west central regions (Gujrat, Madhya Pradesh, Chhattisgarh, Uttar Pradesh, and Bihar) and some parts of southeast and northeast India (Assam, Meghalaya, Tripura) show low coverage of exclusive use of hygienic period products during menstruation (low-low zone). Only 7 districts have high to low coverage of exclusive use of hygienic period products during menstruation. The significance map in Fig. 7b shows that a total of 327 districts in India have significant neighbouring effects of exclusive use of hygienic period products during menstruation, with 83 districts being significant at p-0.001, and 122 districts each being significant at p-0.01 and p-0.05. Furthermore, the Moran's I scatter plot in Fig. 7c indicates a high degree of positive spatial autocorrelation with a value of 0.72. This suggests that there may be underlying spatial patterns or factors influencing the distribution of menstrual hygiene practices across different districts in India. The scatter plot shows a strong clustering of values, indicating that districts with high rates of exclusive use of hygienic period products during menstruation are spatially clustered together, and so are districts with low rates of exclusive use of hygienic period products.

### Rural–urban differentials in exclusively using hygienic period products, India

Table 2 presents the results of a multivariate decomposition analysis examining factors influencing the adoption of hygienic period products during menstruation in India. Household wealth emerges as the most significant contributor, explaining 49.25% of the observed gap due to differences in characteristics. Specifically, the poorer (− 7.36%) and middle (− 2.65%) wealth quintiles show negative contributions, indicating higher usage rates among rural women in these groups compared to their urban counterparts. In contrast, the richer (15.28%) and richest (43.98%) wealth quintiles contribute positively, reflecting greater adoption among urban women in these categories. Women with no education contribute positively (13.41%) to the rural–urban gap, suggesting lower usage rates among uneducated rural women. Conversely, those with primary (-0.54%) and secondary (− 4.73%) education levels contribute negatively, indicating higher rural usage rates among these groups. Higher education levels contribute positively (18.68%), showing increased adoption among urban women. Access to media at least once a week significantly boosts usage (10.73%), while less frequent access (− 2.76%) associates with lower adoption rates among rural women. Regionally, the Central (4.02%), South (1.96%), East (1.05%), and Northeast (0.77%) regions contribute positively, highlighting disparities in menstrual hygiene practices. Conversely, the West region shows a negative contribution (− 2.83%), indicating barriers to usage. Women who attend cinema or theatre monthly also contribute positively (3.26%), suggesting social engagement promotes hygienic practices. Age-wise, women aged 20–24 (− 0.26%) exhibit slightly lower usage rates than those aged 15–19. Among religious groups, Muslim women (− 1.97%) show negative contributions relative to Hindu women. Toilet facilities, social groups, and discussion of menstrual hygiene with healthcare workers also contribute, though to a lesser extent compared to wealth, education, media access, and regional factors.

**Table 2:** Multivariate decomposition estimates for Rural–Urban differentials for practicing hygienic period products during menstruation, India, NFHS-5 (2019–2021).

Background characteristics	Due to differences in characteristics					Due to the difference in coefficients				
	Coef	SE	p-value	% Contribution		Coef	SE	p-value	% Contribution	
Age group (in years)										
15–19	0.000	0.000			− 0.26	0.000	0.000			− 2.74
20–24	0.000	0.000	0.000	− 0.26		0.005	0.001	0.001	− 2.74	
Education										
No education	0.000	0.000			13.41	0.000	0.000			− 1.68
Primary	0.001	0.000	0.000	− 0.54		0.000	0.000	0.758	0.05	
Secondary	0.008	0.000	0.000	− 4.73		− 0.001	0.003	0.826	0.44	
Higher	− 0.032	0.001	0.000	18.68		0.004	0.002	0.046	− 2.17	
Religion										
Hindu	0.000	0.000			− 2.17	0.000	0.000			0.56
Muslim	0.003	0.000	0.000	− 1.97		− 0.001	0.001	0.340	0.4	
Christian	0.000	0.000	0.000	0.00		0.000	0.001	0.707	0.11	
Others	0.000	0.000	0.000	− 0.20		0.000	0.000	0.829	0.05	
Social group										
SC	0.000	0.000			1.29	0.000	0.000			4.29
ST	− 0.002	0.000	0.000	1.31		− 0.001	0.001	0.068	0.65	
OBC	0.000	0.000	0.000	− 0.13		− 0.002	0.002	0.264	1.03	
Others	0.000	0.000	0.475	0.11		− 0.004	0.001	0.001	2.61	
Wealth quintile										
Poorest	0.000	0.000			49.25	0.000	0.000			3.91
Poorer	0.013	0.000	0.000	− 7.36		0.000	0.001	0.417	− 0.24	
Middle	0.005	0.000	0.000	− 2.65		0.001	0.001	0.620	− 0.31	
Richer	− 0.026	0.001	0.000	15.28		− 0.001	0.002	0.756	0.34	
Richest	− 0.075	0.001	0.000	43.98		− 0.007	0.003	0.011	4.12	
Type of toilet										
Flush	0.000	0.000			2.63	0.000	0.000			0.20
Pit/dry	0.000	0.000	0.898	0.01		0.000	0.000	0.676	0.07	
No facility/openspacec	− 0.005	0.000	0.000	2.92		0.000	0.000	0.669	0.07	
Others	0.001	0.000	0.011	− 0.30		0.000	0.000	0.736	0.06	
Access to media										
Not at all	0.000	0.000			7.97	0.000	0.000			− 2.59
Less than once a week	0.005	0.000	0.000	− 2.76		0.001	0.001	0.087	− 0.82	
At least once a week	− 0.018	0.001	0.000	10.73		0.003	0.003	0.315	− 1.77	
Usually go to cinema/theatre at least once in a month										
No	0.000	0.000			3.26	0.000	0.000			− 0.28
Yes	− 0.006	0.000	0.000	3.26		0.000	0.001	0.593	− 0.28	
Discussed menstrual hygiene with the healthcare worker										
No	0.000	0.000			− 0.18	0.000	0.000			− 0.17
Yes	0.000	0.000	0.000	− 0.18		0.000	0.000	0.075	− 0.17	
Region										
North	0.000	0.000			4.97	0.000	0.000			− 6.09
Central	− 0.007	0.000	0.000	4.02		0.001	0.001	0.327	− 0.56	
East	− 0.002	0.000	0.000	1.05		0.001	0.001	0.025	− 0.84	
Northeast	− 0.001	0.000	0.000	0.77		0.001	0.001	0.028	− 0.87	
West	0.005	0.000	0.000	− 2.83		0.004	0.001	0.000	− 2.33	
South	− 0.003	0.000	0.000	1.96		0.003	0.001	0.028	− 1.49	

Coef.: decomposition coefficients; SE: standard error; % Contribution: percentage contribution of each variable category to the overall rural–urban gap in practicing hygienic methods during menstruation.

## Discussion

The study sheds light on critical insights concerning MHH among young women in India. Our findings indicate that despite the recognized importance of effective MHH, a substantial proportion of young women aged 15 to 24 still face challenges in accessing hygienic menstrual protection. Specifically, our study reveals that only 76.15% of young women in India have access to such protection, with notable regional disparities observed. While the South and North regions exhibit relatively higher coverage rates (90.765% and 87.83%, respectively), the Northeast, Central, and Eastern regions lag behind, with coverage rates ranging from 69.13 to 71.43%. Government interventions, such as the Rashtriya Kishore Swasthya Karyakram (RKSK) and initiatives aimed at distributing free sanitary napkins, have undoubtedly contributed to enhancing MHH. However, it is essential to acknowledge that progress in this area may be influenced by various socio-economic factors and the level of education among young women. Increased education levels tend to correlate with heightened awareness of MHH's significance and improved access to affordable menstrual hygiene products. Moreover, the implementation of government schemes like the RKSK, launched in 2014 to educate adolescent girls about menstruation and facilitate access to sanitary pads, has significantly contributed to advancing MHH in India^45^. The study by Kathuria and Sherin Raj based on NFHS 4 (2015–2016) data, found that 57% of women were using hygienic period products during menstruation in India. The Central and Eastern regions had a usage rate of 44%, while the Southern region had an 80% usage rate^46^. The government schemes have helped in improving the situation, such as the distribution of free sanitary napkins in schools by state governments in Uttar Pradesh, Rajasthan, Maharashtra, Odisha, Chhattisgarh, Andhra Pradesh, and Kerala^32,47^. Additionally, recent policy interventions, including the Mukhyamantri Kishori Swasthya Yojana (MKSY), have aimed to address MHH challenges by providing financial assistance to adolescent girls for purchasing sanitary pads. For instance, the MKSY initiative in Bihar offers direct benefit transfers (DBT) of Rs 300 annually to female students in Classes 7 to 12, accompanied by provisions for menstrual leave for female employees^28,48,49^. Furthermore, the heightened attention to menstrual health issues at the national level, as exemplified by Prime Minister Narendra Modi's remarks in August 2020, underscores the growing recognition of MHH as a public health imperative^50^. Recent state-level initiatives, such as the introduction of monthly awareness classes for students in Andhra Pradesh, further reinforce the commitment to addressing menstrual health concerns^51^.

Despite these advancements, the persistent stigma surrounding menstruation continues to pose significant challenges for women, adversely impacting their health and educational outcomes. Government schemes exist, but there are still gaps in menstrual hygiene that need greater and more nuanced attention. Neglecting proper hygiene practices during menstruation can lead to various health problems such as cervical cancer, reproductive tract infections, Hepatitis B infection, yeast infections, and urinary tract infections. In more severe cases, toxic shock syndrome can even lead to death^52,53^. Moreover, the lack of access to clean toilets and sanitary products in schools contributes to school dropout rates among girls, particularly in rural areas^54^. Our study highlights the role of household wealth, education, and access to media in shaping the rural–urban gap in the use of hygienic period products during menstruation. Addressing this gap requires multifaceted interventions aimed at increasing family income, enhancing medical infrastructure, and improving media access in rural areas. Moreover, efforts to raise awareness and destigmatize menstruation are essential to promoting positive menstrual health outcomes across diverse socio-economic contexts^4,32^.

The NFHS-5 report reveals that 20.71% of rural women in India lack access to proper toilet facilities, compelling them to resort to open defecation, thereby posing challenges for menstrual hygiene, particularly in using sanitary napkins^55^. Conversely, our study suggests a relatively minimal rural–urban gap of 2.63% in terms of access to toilets for menstrual hygiene. However, caution must be exercised in interpreting this finding, as it may not entirely reflect the efficacy of government sanitation efforts. While NFHS-4 data from 2015 to 2016 revealed that 42.40% of women did not utilize hygienic period products during menstruation, this figure decreased significantly to 23.85% in NFHS-5 (2019–2021). Similarly, the proportion of households lacking toilet access decreased from 39% to 18.08% over the same period^55^. Although these improvements imply positive outcomes potentially attributable to governmental initiatives aimed at sanitation improvement and open defecation eradication, it is essential to acknowledge the limitations of solely relying on toilet access as an indicator of menstrual hygiene improvement. The observed decrease in the lack of toilet facilities does not inherently equate to a corresponding improvement in menstrual hygiene practices. Literature suggests that the mere provision of toilets does not guarantee their utilization, echoing the distinction between constructing latrines and actual latrine use. This underscores the importance of considering broader socio-cultural, economic, and behavioural factors shaping menstrual hygiene practices. Therefore, while government initiatives have shown progress in enhancing sanitation and hygiene practices, a nuanced understanding of the underlying factors is crucial for devising effective, sustainable interventions to bridge the rural–urban gap and ensure holistic advancements in menstrual hygiene management.

Our study found that highly educated women have a 5.38 times higher likelihood of using hygienic period products during menstruation, which is supported by previous literature^32,46^. In order to address concerns about gender discrimination and women's empowerment in the country, the Government of India introduced the Beti Bachao Beti Padhao (BBBP) scheme in 2015. The name Beti Bachao Beti Padhao translates to 'Save the girl child, educate the girl child'^56^. The scheme aims to raise awareness against gender bias and improve the effectiveness of welfare services for girls. Initially launched with funding of Rs. 100 crore (US$ 13.5 million), the scheme needs to expand its funding to support education on menstrual health and hygiene for highly educated women^57^.

Based on the findings of our study and existing literature, it is evident that addressing menstrual hygiene management (MHH) in India requires multifaceted policy interventions that prioritize education, access to resources, and socio-cultural empowerment. Firstly, there is a need for comprehensive menstrual health education programs targeting both urban and rural areas. These programs should be integrated into school curricula from an early age to ensure that both girls and boys are educated about menstrual hygiene, breaking the stigma surrounding menstruation and promoting gender equality. Additionally, expanding the scope of government schemes like the Rashtriya Kishore Swasthya Karyakram (RKSK) and the Beti Bachao Beti Padhao (BBBP) scheme to include menstrual health education and access to hygienic products for highly educated women can further promote awareness and empowerment. Secondly, improving access to affordable and sustainable menstrual hygiene products is essential, particularly in rural areas. This can be achieved through initiatives such as subsidizing the cost of sanitary napkins and menstrual cups, promoting the use of eco-friendly alternatives, and establishing distribution channels in remote regions. Thirdly, infrastructure development initiatives like the Swachh Bharat Mission (SBM) and the Pradhan Mantri Sauchalay Yojana (PMSY) should continue to prioritize the construction of sanitation facilities, particularly in schools and public spaces, to ensure dignity and privacy for women and girls. Finally, community engagement and participation are crucial for the success of MHH initiatives. Engaging local stakeholders, including community leaders, NGOs, and women's self-help groups, can facilitate the design and implementation of culturally sensitive programs tailored to the specific needs of different regions and communities. By adopting a holistic approach that addresses education, access, infrastructure, and community involvement, policymakers can effectively promote menstrual health and hygiene, empower women, and advance gender equity in India.

## Conclusion

This study highlights the low usage of hygienic period products among young women in India and significant regional disparities in the exclusive use of such methods. The central districts of India were found to have considerably low usage of hygienic period products. While the improvement of menstrual hygiene and health has been discussed in policy circles, there has been little attention paid to regional variations in patterns of use. Future interventions and programs aimed at enhancing menstrual hygiene among young women must therefore focus on reducing geographical disparities in India.

To address the issue, policymakers and stakeholders must create awareness campaigns at the grassroots level, promoting the importance of sanitary napkins for a healthy life through advertisements and other means. The cost of sanitary napkins remains a significant barrier in India, with the average cost of Rs. 48 per napkin being too expensive for many Indian women, especially those from poor families^58^. To address this issue, the government should provide training to Self Help Groups to produce sanitary napkins at minimum cost and sell them at an affordable price in the market, making them accessible to all women.

## Data Availability

The dataset analyzed in the current study is available online on the official website of the Demographic Health Survey Program https://www.dhsprogram.com. However, the datasets used can be made available from the corresponding author upon reasonable request.

## References

[1] Anton, B., Kim, W., Nair, A. & Wang, E. *Menstrual Hygiene Management-Evidence from the 6th Round of MICS*. 11 (Data Anal Sect Div Data, Anal Plan Monit UNICEF New York, 2021).

[2] Roeckel, S., Cabrera-Clerget, A. & Yamakoshi, B. *Guide to Menstrual Hygiene Materials* 6–36 (UNICEF, 2019).

[3] Mudey, A. B., Kesharwani, N., Mudey, G. A. & Goyal, R. C. A cross-sectional study on awareness regarding safe and hygienic practices amongst school going adolescent girls in rural area of Wardha District, India. *Glob. J. Health Sci.* **2**(2), 225 (2010). 10.5539/gjhs.v2n2p225

[4] Kaur, R., Kaur, K. & Kaur, R. Menstrual hygiene, management, and waste disposal: Practices and challenges faced by girls/women of developing countries. *J. Environ. Public Health.* **2018**, 1730964 (2018). 29675047 10.1155/2018/1730964 PMC5838436

[5] Chandra-Mouli V, Patel SV. Mapping the knowledge and understanding of menarche, menstrual hygiene and menstrual health among adolescent girls in low-and middle-income countries. *Palgrave Handb. Crit. Menstruation Stud.* 609–636 (2020). 33347167

[6] Hennegan, J. & Montgomery, P. Do menstrual hygiene management interventions improve education and psychosocial outcomes for women and girls in low and middle income countries? A systematic review. *PLoS ONE* **11**(2), e0146985 (2016). 26862750 10.1371/journal.pone.0146985 PMC4749306

[7] El-Gilany, A.-H., Badawi, K. & El-Fedawy, S. Menstrual hygiene among adolescent schoolgirls in Mansoura, Egypt. *Reprod. Health Matters.* **13**(26), 147–152 (2005). 16291496 10.1016/S0968-8080(05)26191-8

[8] Khanna, A., Goyal, R. S. & Bhawsar, R. Menstrual practices and reproductive problems: A study of adolescent girls in Rajasthan. *J. Health Manag.* **7**(1), 91–107 (2005). 10.1177/097206340400700103

[9] Chauhan, S. *et al.* Examining the predictors of use of sanitary napkins among adolescent girls: A multi-level approach. *PLoS ONE.* **16**(4), e0250788 (2021). 33930035 10.1371/journal.pone.0250788 PMC8087036

[10] Sharma, S., Mehra, D., Brusselaers, N. & Mehra, S. Menstrual hygiene preparedness among schools in India: A systematic review and meta-analysis of system-and policy-level actions. *Int. J. Environ. Res. Public Health.* **17**(2), 647 (2020). 31963862 10.3390/ijerph17020647 PMC7013590

[11] Sommer, M. & Sahin, M. Overcoming the taboo: Advancing the global agenda for menstrual hygiene management for schoolgirls. *Am. J. Public Health.* **103**(9), 1556–1559 (2013). 23865645 10.2105/AJPH.2013.301374 PMC3780686

[12] Kyilleh, J. M., Tabong, P.T.-N. & Konlaan, B. B. Adolescents’ reproductive health knowledge, choices and factors affecting reproductive health choices: A qualitative study in the West Gonja District in Northern region, Ghana. *BMC Int. Health Hum. Rights.* **18**(1), 1–12 (2018). 29361947 10.1186/s12914-018-0147-5 PMC5782392

[13] Rani, P. Knowledge and practices of menstrual hygiene among married adolescents and young women in Chittoor district of Andhra Pradesh: India. *J. Nurs. Health Sci.* **3**(2), 6–15 (2014).

[14] Kansal, S., Singh, S. & Kumar, A. Menstrual hygiene practices in context of schooling: A community study among rural adolescent girls in Varanasi. *Indian J. Community Med. Off. Publ. Indian Assoc. Prev. Soc. Med.* **41**(1), 39 (2016). 10.4103/0970-0218.170964 PMC4746952 26917872

[15] Shanbhag, D. *et al.* Perceptions regarding menstruation and practices during menstrual cycles among high school going adolescent girls in resource limited settings around Bangalore city, Karnataka, India. *Int. J. Collab. Res. Intern. Med. Public Health* **4**(7), 1353 (2012).

[16] Sharanya, T. Reproductive health status and life skills of adolescent girls dwelling in slums in Chennai, India. *Natl. Med. J. India.* **27**(6), 305–310 (2014). 26133325

[17] Ramachandra, K., Gilyaru, S., Eregowda, A. & Yathiraja, S. A study on knowledge and practices regarding menstrual hygiene among urban adolescent girls. *Int. J. Contemp. Pediatr.* **3**(1), 142–145 (2016). 10.18203/2349-3291.ijcp20160147

[18] Thakur, H. *et al.* Knowledge, practices, and restrictions related to menstruation among young women from low socioeconomic community in Mumbai, India. *Front. Public Health* **2**, 72 (2014). 25072044 10.3389/fpubh.2014.00072 PMC4080761

[19] Muralidharan, A., Patil, H. & Patnaik, S. Unpacking the policy landscape for menstrual hygiene management: Implications for school Wash programmes in India. *Waterlines.* **34**, 79–91 (2015). 10.3362/1756-3488.2015.008

[20] Hennegan, J. *Understanding Interventions to Improve Menstrual Health in Low and Middle Income Countries: Evidence and Future Directions* (University of Oxford, 2017).

[21] Babbar, K., Martin, J., Ruiz, J., Parray, A. A. & Sommer, M. Menstrual health is a public health and human rights issue. *Lancet Public Health* **7**(1), e10–e11 (2022). 34717798 10.1016/S2468-2667(21)00212-7 PMC8552814

[22] Sommer, M., Utami, D. & Gruer, C. Menstrual hygiene management considerations during Ebola response: A qualitative exploration. *J. Int. Humanit. Action.* **7**(1), 19 (2022). 10.1186/s41018-022-00128-9

[23] Dars, S., Sayed, K. & Yousufzai, Z. Relationship of menstrual irregularities to BMI and nutritional status in adolescent girls. *Pak. J. Med. Sci.* **30**(1), 141 (2014). 24639848 10.12669/pjms.301.3949 PMC3955559

[24] Jogdand, K. & Yerpude, P. A community based study on menstrual hygiene among adolescent girls. *Indian J. Matern. Child Health* **13**(3), 1–6 (2011).

[25] Guterres, A. Sustainable Development Goals Report 2020 | United Nations. (2020) Accessed 9 May 2023, https://www.un.org/en/desa/sustainable-development-goals-report-2020

[26] Geertz, A., Iyer, L., Kasen, P., Mazzolar, F. & Peterson, K. *Menstrual Health in India: An Update* (2016).

[27] Vishwakarma, D., Puri, P. & Sharma, S. K. Interlinking menstrual hygiene with Women’s empowerment and reproductive tract infections: Evidence from India. *Clin. Epidemiol. Glob. Health* **10**, 100668 (2021). 10.1016/j.cegh.2020.11.001

[28] Ram, U., Pradhan, M. R., Patel, S. & Ram, F. Factors associated with disposable menstrual absorbent use among young women in India. *Int. Perspect. Sex Reprod. Health.* **46**, 223–234 (2020). 33108760 10.1363/46e0320

[29] Roy, A. *et al.* Prevalence and correlates of menstrual hygiene practices among young currently married women aged 15–24 years: An analysis from a nationally representative survey of India. *Eur. J. Contracept. Reprod. Health Care.* **26**(1), 1–10 (2021). 32938257 10.1080/13625187.2020.1810227

[30] Kathuria, B. & Raj, S. Effects of socio-economic conditions on usage of hygienic method of menstrual protection among young women in EAG states of India. *Amity J. Healthc. Manag.* **3**(1), 40–52 (2018).

[31] Hema Priya, S. *et al.* A study of menstrual hygiene and related personal hygiene practices among adolescent girls in rural Puducherry. *Int. J. Community Med. Public Health* **4**(7), 2348–2355 (2017). 10.18203/2394-6040.ijcmph20172822

[32] Singh, A. *et al.* Menstrual hygiene practices among adolescent women in rural India: A cross-sectional study. *BMC Public Health.* **22**(1), 1–18 (2022). 36401238 10.1186/s12889-022-14622-7 PMC9675161

[33] Singh, A., Chakrabarty, M., Chowdhury, S. & Singh, S. Exclusive use of hygienic menstrual absorbents among rural adolescent women in India: A geospatial analysis. *Clin. Epidemiol. Glob. Health* **17**, 101116 (2022). 10.1016/j.cegh.2022.101116

[34] Babbar, K. & Garikipati, S. What socio-demographic factors support disposable vs. sustainable menstrual choices? Evidence from India’s National Family Health Survey-5. *PLoS ONE.* **18**(8), e0290350 (2023). 37590271 10.1371/journal.pone.0290350 PMC10434932

[35] Singh, A. & Chakrabarty, M. Spatial heterogeneity in the exclusive use of hygienic materials during menstruation among women in urban India. *PeerJ.* **11**, e15026 (2023). 36967987 10.7717/peerj.15026 PMC10035429

[36] Chakrabarty, M., Singh, A., Let, S. & Singh, S. Decomposing the rural–urban gap in hygienic material use during menstruation among adolescent women in India. *Sci. Rep.* **13**(1), 22427 (2023). 38104217 10.1038/s41598-023-49682-1 PMC10725416

[37] Babbar, K., Vandana, & Arora, A. Bleeding at the margins: Understanding period poverty among SC and ST women using decomposition analysis. *J. Dev. Stud.* **60**(1), 131–146 (2024). 10.1080/00220388.2023.2252139

[38] Babbar, K., Saluja, D. & Sivakami, M. How socio-demographic and mass media factors affect sanitary item usage among women in rural and urban India. *Waterlines* **40**(3), 160–178 (2021). 10.3362/1756-3488.21-00003

[39] Singh, A. *et al.* Wealth-based inequality in the exclusive use of hygienic materials during menstruation among young women in urban India. *PLoS ONE.* **17**(11), e0277095 (2022). 36445854 10.1371/journal.pone.0277095 PMC9707774

[40] Sivakami, M. Menstrual hygiene practices among Indian women. In *Atlas of Gender and Health Inequalities in India*. 127–134 (Springer, 2024).

[41] Meher, T. & Sahoo, H. Secular trend in age at menarche among Indian women. *Sci. Rep.* **14**(1), 5398 (2024). 38443461 10.1038/s41598-024-55657-7 PMC10914750

[42] Meher, T. & Sahoo, H. Dynamics of usage of menstrual hygiene and unhygienic methods among young women in India: A spatial analysis. *BMC Womens Health.* **23**(1), 573 (2023). 37932760 10.1186/s12905-023-02710-8 PMC10629021

[43] Singh, A., Chakrabarty, M., Chandra, R., Chowdhury, S. & Singh, S. Intra-urban differentials in the exclusive use of hygienic methods during menstruation among young women in India. *PLoS Glob. Public Health* **3**(6), e0002047 (2023). 37310954 10.1371/journal.pgph.0002047 PMC10263343

[44] Khan, J. & Mohanty, S. K. Spatial heterogeneity and correlates of child malnutrition in districts of India. *BMC Public Health.* **18**(1), 1–13 (2018). 10.1186/s12889-018-5873-z PMC6098604 30119652

[45] Barua, A., Watson, K., Plesons, M., Chandra-Mouli, V. & Sharma, K. Adolescent health programming in India: A rapid review. *Reprod. Health.* **17**(1), 1–10 (2020). 32493471 10.1186/s12978-020-00929-4 PMC7271491

[46] Kathuria, B. & Sherin Raj, T. P. Factors explaining regional variations in menstrual hygiene practices among young women in India: Evidence from NFHS-4. *J. Soc. Health* **5**, 35–38 (2022).

[47] Chakrabarty, M., Singh, A., Singh, S. & Tripathi, P. Spatiotemporal change in socioeconomic inequality in hygienic menstrual product use among adolescent girls in India during 2015–2019. *Int. J. Equity Health.* **22**(1), 202 (2023). 37773141 10.1186/s12939-023-02020-3 PMC10543847

[48] Singh, N. & Singh, L. Descriptive study of menstrual health management practices in India. *Think India J.* **22**(4), 8141–8151 (2019).

[49] Rajput, S. & Jain, P. Much needed ‘pad man’ for Indian females to be dignified: A case study on period poverty. In *Social and Sustainability Marketing* 647–718 (Productivity Press, 2021).

[50] Crawford, B. J. & Waldman, E. G. Period poverty in a pandemic: Harnessing law to achieve menstrual equity. *Wash UL Rev.* **98**, 1569 (2020).

[51] Jha, B. Menstrual health schemes in India: Much done, much still to be accomplished. SWARAJYA. (2022) Accessed 12 May 2023, https://swarajyamag.com/blogs/why-india-needs-a-well-planned-menstrual-health-scheme.

[52] Abebe, M., Eshetie, S. & Tessema, B. Prevalence of sexually transmitted infections among cervical cancer suspected women at University of Gondar Comprehensive Specialized Hospital, North-west Ethiopia. *BMC Infect. Dis.* **21**(1), 1–7 (2021). 33888090 10.1186/s12879-021-06074-y PMC8063310

[53] Rawat, G., Tyagi, A., Saxena, C. Menstrual hygiene: A salubrious approach to curb gynecological problems. *Women’s Health Care Anal.* **1**(1) (2020).

[54] Dasra KT& U. Spot On!: Improving Menstrual Management in India (2015) https://www.dasra.org/resource/improving-menstrual-health-and-hygiene.

[55] NFHS-5. National Family Health Survey (NFHS-5) 2019–21. Int Inst Popul Sci 1–116 (2021) http://rchiips.org/nfhs/factsheet_NFHS-5.

[56] Rani, J., Dahiya, M. & Yadav, B. Awareness regarding Beti Bachao Beti Padhao scheme in Rewari districts. *Int. J. Curr. Microbiol. Appl. Sci.* **8**, 2299–2305 (2019). 10.20546/ijcmas.2019.807.280

[57] Nigam, A. K., Srivastava, R., Kulshreshtha, A. C. & Kumar, K. *Female Empowerment: A Life-Cycle Analysis* (Cambridge Scholars Publishing, 2020).

[58] Garg, R., Goyal, S. & Gupta, S. India moves towards menstrual hygiene: Subsidized sanitary napkins for rural adolescent girls—issues and challenges. *Matern. Child Health J.* **16**, 767–774 (2012). 21505773 10.1007/s10995-011-0798-5

